# The Indication of Poor Prognosis by High Expression of ENO1 in Squamous Cell Carcinoma of the Lung

**DOI:** 10.1155/2021/9910962

**Published:** 2021-08-30

**Authors:** Wan-Ying Huang, Gang Chen, Shang-Wei Chen, Yi-Wu Dang, Yun Deng, Hua-Fu Zhou, Jin-Liang Kong, Yu Zhang, Jun-Xian Mo, Chang-Bo Li, Juan He

**Affiliations:** ^1^Department of Pathology, The First Affiliated Hospital of Guangxi Medical University, No. 6, Shuangyong Road, Nanning, Guangxi, China; ^2^Department of Cardio-Thoracic Surgery, The First Affiliated Hospital of Guangxi Medical University, No. 6, Shuangyong Road, Nanning, Guangxi, China; ^3^Ward of Pulmonary and Critical Care Medicine, Department of Respiratory Medicine, The First Affiliated Hospital of Guangxi Medical University, No. 6, Shuangyong Road, Nanning, Guangxi, China; ^4^Department of Pathology, Shandong Provincial Hospital Affiliated to Shandong First Medical University, Jinan, China; ^5^Department of Cardio-Thoracic Surgery, The Seventh Affiliated Hospital of Guangxi Medical University, Wuzhou Gongren Hospital, Wuzhou, No. 1, Gaodi Road, Guangxi Zhuang Autonomous Region 543000, China

## Abstract

The purpose of this study is to investigate the significance of alpha-enolase (ENO1) expression in squamous cell carcinoma of the lung (LUSC), its prognostic value, and prospective molecular mechanism. Using multiplatforms data, including in-house immunohistochemistry, in-house real-time fluorescence quantitative polymerase chain reaction (RT-qPCR), in-house microarray, and public high-throughput data, the expression significance and prognostic role of ENO1 in LUSC tissues were analyzed comprehensively. With the combination of all eligible cases, compared with 941 non-LUSC lung tissues, ENO1 was significantly overexpressed in 1163 cases of LUSC (standardized mean difference (SMD) = 1.23, 95% confidence interval (CI) = 0.76–1.70, *P* < 0.001). ENO1 also displayed a great ability to differentiate LUSC tissues from non-LUSC lung tissues (AUC = 0.8705) with the comprehensive sensitivity being 0.88 [0.83–0.92], and comprehensive specificity being 0.89 [0.84–0.94]). Moreover, in 1860 cases of LUSC with survival information, patients with higher expression of ENO1 had poorer prognosis (hazard ratio (HR) = 1.20, 95% CI = 1.01–1.43, *P* = 0.043). ENO1 and its related genes mainly participated in the pathways of cell division and proliferation. In conclusion, the upregulation of ENO1 could affect the carcinogenesis and unfavorable outcome of LUSC.

## 1. Introduction

Lung carcinoma remained a contributory factor in human death [1], with 85% of lung cancers classified as non-small cell lung carcinoma (NSCLC) [1]. One major subtype of NSCLC was considered squamous cell carcinoma of the lung (LUSC), which was lethally invasive [2, 3]. Its incidence rate remained the highest among the subtypes of lung cancers until 2015 [4]. Nevertheless, its proportion in lung carcinoma was reduced to 35% after 2015 [2, 4]. In addition to smoking, the development of LUSC was also linked to abnormal gene expression as well as mutated driver gene [2]. So far, the research plateaued regarding the molecularly targeted therapy for LUSC. Hence, it was of great benefit for the prognosis of patients and clinical studies to discover a novel tumor marker.

Alpha-enolase (ENO1) worked as an enzyme that had a pivotal role in glycolysis, particularly in the glycolysis of tumor cells [5]. Its overexpression and posttranslational modification were reported to exert carcinogenic effects in a wide range of tumors, including retinoblastoma [6], glioma [7], pancreatic cancer [8], bladder cancer [9], colorectal cancer [10], and hepatocellular carcinoma [11].

In six studies, the expression of ENO1 was observed to be upregulated or downregulated in lung cancer tissues, which suggested that the clinical influence of ENO1 in lung cancer tissues remained obscure. Five of them showed that the expression of ENO1 was increased in lung cancer by immunohistochemistry and real-time fluorescence quantitative polymerase chain reaction (RT-qPCR) [12–16]. On the contrary, Chang et al. [17] used immunohistochemical staining to observe the low-level expression of ENO1 in NSCLC tissues. In addition to the inconsistent results of the expression of ENO1 in lung cancer, the prognostic effect of ENO1 on tumors is also opposite. In addition, some studies have reported that the prognostic effect of ENO1 in various cancers was opposite. According to reports, patients with tumors with high expression of ENO1 have a poor prognosis, including NSCLC [14, 18], breast cancer [19], glioma [7], bladder cancer [9], pancreatic cancer [20], liver cancer [21], and gastric cancer [21]. Interestingly, Chang et al. [17] showed that the prognosis of patients with NSCLC with downregulated expression of ENO1 was poor.

These conflicting findings might be attributed to single research technique and small samples. Currently, only one study by Zhang et al. [12] explained the expression of ENO1 in LUSC, however, providing no information regarding the ENO1 mRNA. Most importantly, little research reported the prognostic value of ENO1 in LUSC. Consequently, this study would holistically analyze the expression of ENO1 in LUSC and its clinically prognostic capability by varied research approaches, including immunohistochemical staining, RT-qPCR, microarray of our hospital, as well as public high-throughput data.

## 2. Materials and Methods

### 2.1. Collection of Clinical Samples

Three samples of freshly resected LUSC tissues and corresponding adjacent noncancerous lung tissues were collected from Department of Thoracic Surgery of the First Hospital Affiliated to Guangxi Medical University between August 2017 and October 2017. The fresh tissues were converted to microarray. Also, Department of Pathology provided twelve samples of paraffin-embedded LUSC tissues and corresponding adjacent normal tissues as controls from December 2018 to February 2019. These samples were processed with immunohistochemical staining and RT-qPCR testing. All the patients involved in this study had not received any treatment prior to the operation. The experiment was approved by Ethics Committee of the First Hospital Affiliated to Guangxi Medical University. Informed content had been obtained from each patient or his/her relatives. The schematic diagrams are shown in Supplementary Figure 1.

### 2.2. Immunohistochemistry (IHC)

Immediately after resection, the tissue samples were treated with fixation (10%, neutral buffered formalin) for 24 hours, and then the tissues were dehydrated and paraffin-embedded. These paraffin-embedded tissues were subsequently sliced (4 *µ*m in size) for IHC staining. The IHC staining process was elaborately explained in previous studies [22–25]. ENO1 antibodies were purchased from Abcam (Cambridge, UK). A total of 10 microscope fields of view were randomly selected, and the marking was based on the percentage of positive cells and staining intensity in the fields. In terms of the average percentage of positive cells, the marking was as follows: 0 (10%), 1 (10–25%), 2 (26–50%), 3 (51–75%), and 4 (76–100%); according to the staining intensity, 0 was for no staining; 1, for weak staining; 2, for moderate staining; and 3, for strong staining. When the product of average percentage of positive cells and staining intensity was more than 2, the positivity was ascertained. The differences between LUSC tissues and the adjacent noncancerous tissues were calculated by Pearson's chi-squared test. The differential expression of ENO1 in LUSC tissues and non-LUSC lung tissues was tested and verified on the website The Human Protein Atlas (HPA, https://www.proteinatlas.org/).

### 2.3. In-House RT-qPCR and In-House Microarray

From Paraffin-embedded tissues of LUSC, the RNA was extracted according to the instruction of R6954FFPE RNA Kit from OMEGA. The RNA was reversely transcribed in accordance with the instruction of miRcute Plus miRNA First-Strand cDNA Kit (KR211), which was purchased from TIANGEN. The extraction and correction of ENO1 mRNA were described in previous studies [26]. ACTB functioned as the internal reference/control gene [27]. Primer Premier was applied for the design of primers, of which sequence is listed in Table 1. The relative quantification of the expression in each cohort was calculated by threshold cycle value (Ct): ΔCt = Ct value of ENO1 − Ct value of ACTB. 2^−ΔCt^ reflected the expression level of each sample.

### 2.4. Integration Analysis of the Expression of ENO1 mRNA

From HPA, the researchers downloaded the IHC results of ENO1 expression in LUSC and normal lung tissues. Besides, the expression of LUSC mRNA and the clinical information of patients were downloaded from public databases. The searching strategy was referred to previous studies [23, 28–31]. This study entailed databases such as Genotype-Tissue Expression (GTEx, http://genome.ucsc.edu/), The Cancer Genome Atlas database (TCGA, http://genome.ucsc.edu/), Gene Expression Omnibus (GEO, https://www.ncbi.nlm.nih.gov/geo/query/acc.cgi), ArrayExpress, Sequence Read Archive (SRA), Oncomine, PubMed, and Google Scholar, China National Knowledge Infrastructure (CNKI), Chongqing VIP, and Chinese WanFang. The standards for inclusion and exclusion of datasets and the literature on this topic were elaborated in previous research [23]. The included datasets and the literature should fulfill the necessary condition that at least 3 cases of experimental and control samples were involved in the study, and these datasets and the literature had to include the expression data of ENO1 mRNA. Those datasets and the literature that failed to meet the condition were all disregarded. To minimize the batch effect, the datasets on the same platform were integrated. The process of the search of public databases is illustrated in Supplementary Figure 2. The expression data of ENO1 mRNA were extracted from in-house RT-qPCR, in-house microarray, public high throughput databases and literature studies.

### 2.5. The Expression of ENO1 in LUSC and Its Correlation with the Prognosis

From the datasets included in our study, the researchers acquired statistics regarding the patients' age, sex, TNM staging, survival time, and state of being. Moreover, the relationships between ENO1 expression and the patients' prognosis were explored by means of the high-throughput data. The datasets of prognosis of LUSC should include the datasets of ENO1 expression and at least 3 cases of LUSC samples and involve the LUSC patients' survival time and state of being (Supplementary Figure 2). According to the third quartile (Q3) of the expression value of ENO1, the patients were categorized into two groups: the one with high expression and the other with low expression. The hazard ratio (HR) of each dataset was figured out by STATA 12.0. Integration analysis was eventually performed.

### 2.6. Statistical Methods

Receiver operating characteristic (ROC) curve is a tool that can directly represent the performance of classifiers. GraphPad Prism 8 was used to draw scatter plots and ROC curve, in order to illustrate the capability of ENO1 to distinguish LUSC tissues from adjacent normal tissues. Area under ROC curve (AUC) is a standard of measure, which can be used to distinguish LUSC from non-LUSC lung tissue [32]. In terms of the value of AUC, the marking is as follows: excellent (0.9–1), good (0.8–0.9), fair (0.7–0.8), poor (0.6–0.7), and fail (0.5–0.6). Independent samples *T*-test of IBM SPSS statistics v22.0 was employed to work out the differential expression in LUSC and non-LUSC normal tissues (mean and standard deviation). And then, R software was applied to draw forest plots, funnel plots, summary ROC curves (SROC), comprehensive sensitivity plots, and comprehensive specificity plots. The expression of ENO1 mRNA in LUSC and non-LUSC normal tissues was holistically analyzed. The associations between ENO1 expression and clinical pathological characteristics were probed with independent samples *T*-test, Pearson correlation analysis, and Kaplan–Meier survival analysis. *P* < 0.05 was reckoned to be statistically significant.

### 2.7. Analysis of the Regulatory Effect of ENO1 on Potential Target Genes

In the datasets of LUSC mRNA, the correlated genes with correlation coefficient >0.3 and *P* < 0.05 were identified with the Pearson correlation coefficient of R software. In addition, the differentially expressed genes with logFoldchange >1 and *P*< 0.05 were recognized with LIMMA package. The correlated genes that appeared no less than seven times were combined with the differential genes that occurred no less than five times, and thus the differential genes linked to ENO1 expression were finally obtained. These differential genes related to ENO1 expression were then processed with Gene Ontology (GO) enrichment analysis and Kyoto Encyclopedia of Genes and Genomes (KEGG) biological signal pathway enrichment analysis. The top three pathways of KEGG were analyzed with protein-protein interaction by means of Cytoscape 3.7.1 on String. With the assistance of EcCentricity of CytoHubba, 10 core genes that were most closely interacted with ENO1 protein were automatically recognized. The correlation of ENO1 expression with hub genes and the prognostic value of ENO1 were illustrated by linear scatter plots (made by GraphPad Prism 8.0) and Kaplan–Meier survival curves (survival R package).

## 3. Results

### 3.1. IHC Staining

**Figure 1:**
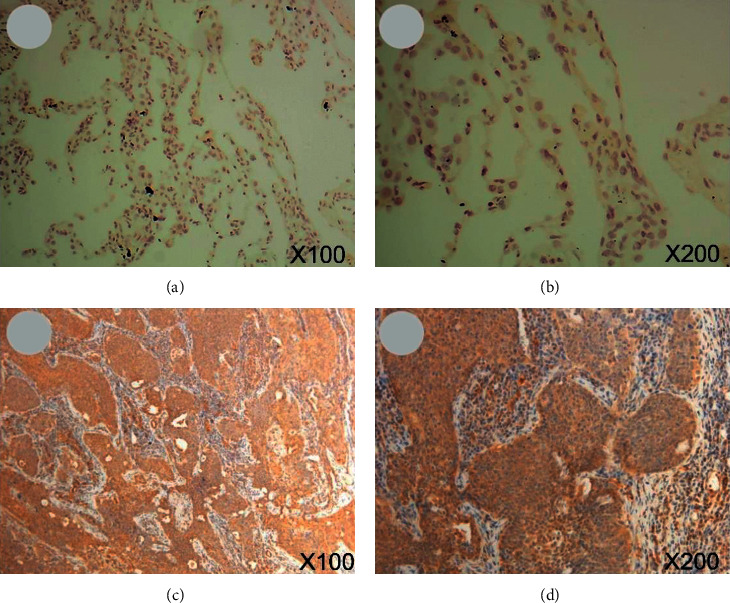
IHC staining of samples from our hospital. (a, b) ENO1 was not stained in normal lung tissues (magnification: 100x and 200x). (c, d) ENO1 was detected in cytoplasmic of LUSC cells, which had medium staining and moderate intensity (magnification: 100x and 200x). Note: IHC, immunohistochemistry; LUSC, squamous cell carcinoma of lung.

**Figure 2:**
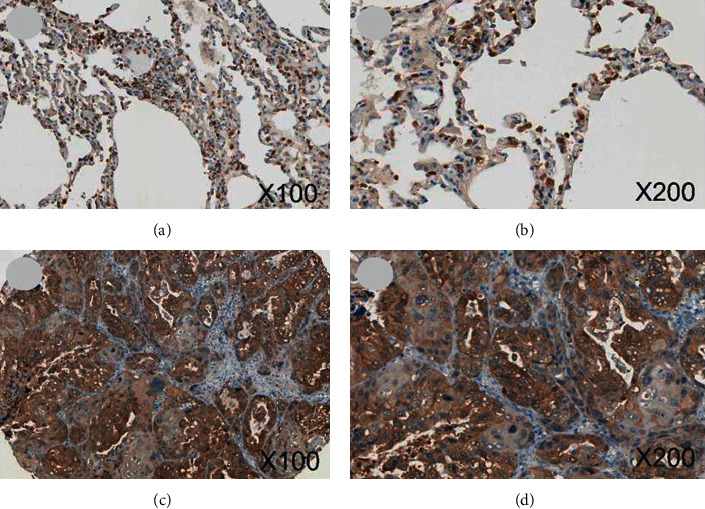
IHC graphs from HPA. (a, b) ENO1 staining was found in cytoblast of normal alveolar epithelial cells (magnification: 100x and 200x). (c, d) ENO1 was detected in cytoplasmic of LUSC cells, which had stronger staining and stronger intensity (magnification: 100x and 200x). Note: HPA, The Human Protein Atlas; IHC, immunohistochemistry; LUSC, squamous cell carcinoma of lung.

ENO1 was observed to display positive expression in five cases of cytoplasm of LUSC (5/12), and its expression was found in one case of normal alveolar epithelial nucleus (1/12) (*X*^2^ = 3.407, *P* = 0.065, Figure 1). The expression pattern of ENO1 in LUSC in HPA database was consistent with the expression pattern of the samples from our hospital (Figure 2). Due to the lack of statistics on LUSC sample size, it was unlikely to conduct statistical analysis on the differential expression of ENO1 in LUSC and adjacent noncancerous tissues.

### 3.2. The Overexpression of ENO1 mRNA in LUSC Tissues according to In-House RT-qPCR and In-House Microarray

**Figure 3:**
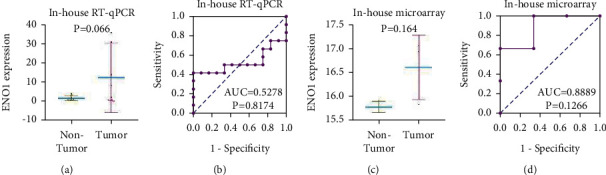
Difference between ENO1 in LUSC and adjacent normal tissue. (a, b) In-house RT-qPCR. (c, d) In-house microarray. According to in-house RT-qPCR and in-house microarray, ENO1 tends to be highly expressed in LUSC tissues ((a) and (c)) and has potential to differentiate LUSC from normal lung tissues ((b) and (d)). Note: ENO1, alpha-enolase; LUSC, squamous cell carcinoma of lung; RT-qPCR, real-time quantitative polymerase chain reaction; AUC, area under curve. *P* < 0.05, statistical significance.

ENO1 tended to exhibit higher expression in LUSC tissues, which was revealed by in-house RT-qPCR and in-house microarray. It was depicted by the scatter plots of in-house RT-qPCR that ENO1 was likely to show higher expression in LUSC tissues than in adjacent nontumor lung tissues (*n* = 12,12.2255 ± 18.0766 vs. 1.5543 ± 1.2613, *P* = 0.066, Figure 3(a)). By the ROC curves of in-house RT-qPCR, ENO1 was less effective in distinguishing LUSC tissues from normal lung tissues (Figure 3(b)). In-house microarray also showed that ENO1 had tendency to exhibit higher expression in LUSC tissues (*n* = 3, 16.6065 ± 0.6714 vs. 15.1879 ± 0.1187, *P* = 0.164 Figure 3(c)). However, ENO1 showed good potential to discern between LUSC and normal lung tissues (AUC = 0.8889, Figure 3(d)).

### 3.3. Integration Analysis of ENO1 Expression in LUSC Tissues

**Figure 4:**
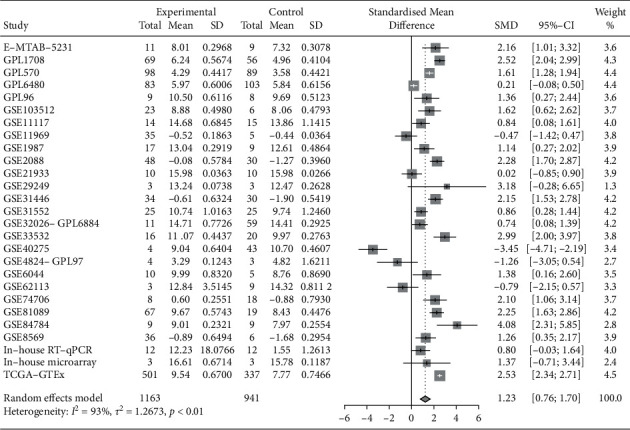
Integration analysis of ENO1 mRNA expression in LUSC. According to in-house RT-qPCR, in-house microarray, public sequencing data, and microarray, the upregulation of ENO1 expression in LUSC tissues was detected. SMD plot, SMD = 1.23 [0.76–1.70], *P* < 0.001. Note: SD, standard deviation; SMD, standardized mean difference; LUSC, squamous cell carcinoma of lung.

Integration analysis was performed regarding the expressions of ENO1 in LUSC samples from our hospital as well as from the published data (Supplementary Table 1). The data of in-house RT-qPCR and in-house microarray demonstrated the ENO1's tendency to show upregulated expression in LUSC. Public and published data, including high-throughput RNA-seq data and microarrays data, reflected that ENO1 showed unusually elevated expression in LUSC (Supplementary Figures 3 and 4). Integration analysis of our hospital's samples data, high-throughput RNA-seq data and microarrays data, which disclosed that the expression of ENO1 was upregulated in LUSC tissues (SMD = 1.23 [0.76–1.70], *P* < 0.001, Figure 4), and ENO1 displayed a great ability to differentiate LUSC tissues from normal lung tissues (AUC = 0.8705, Supplementary Figure 5). Also, ENO1 possessed remarkable specificity and sensitivity when discerning between LUSC tissues and normal tissues (comprehensive sensitivity = 0.88 [0.83–0.92], Supplementary Figure 6; comprehensive specificity = 0.89 [0.84–0.94], Supplementary Figure 7). Nevertheless, noticeable heterogeneity of this research manifested itself in the uneven distribution of dots in funnel plots that represented datasets (Supplementary Figure 8).

### 3.4. The Prognosis Value of ENO1 in LUSC

**Figure 5:**
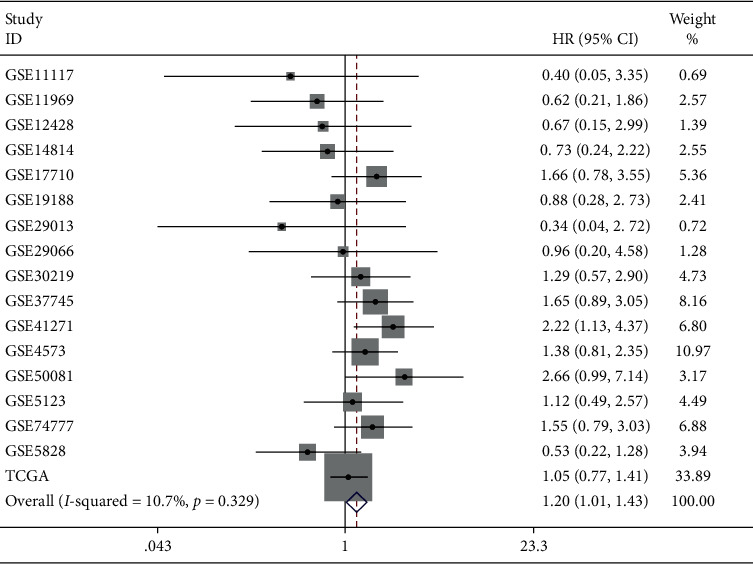
Integration analysis of the effect of high ENO1 expression on the prognosis of LUSC patients. Patients with high ENO1 expression had a poorer prognosis. Comprehensive HR = 1.20 (1.01, 1.43), *P* = 0.043. Note: HR, hazard ratio; LUSC, squamous cell carcinoma of lung.

Based on in-house RT-qPCR, close links were examined between the expression of ENO1 mRNA and T stage as well as staging (Supplementary Table 2). More importantly, patients with higher expression of ENO1 had poorer prognosis, according to the integration analysis of 17 prognosis datasets of LUSC, which included 1860 cases. The overall HR of upregulated ENO1 was 1.20 [1.01–1.43], suggesting that high expression of ENO1 was a risk factor in undesirable survival rate of patients with LUSC (*P* = 0.043, Figure 5).

### 3.5. The Potential Molecular Mechanism of ENO1 in LUSC

**Figure 6:**
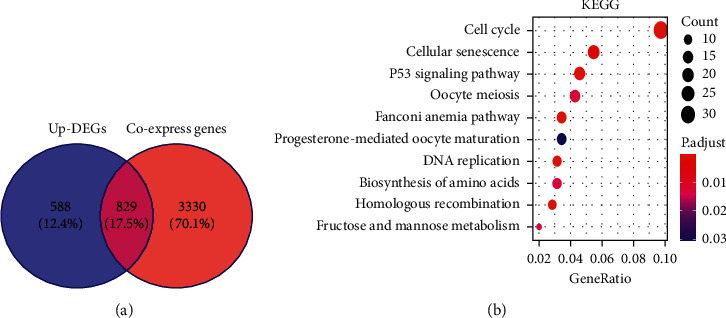
(a) Differentially expressed genes related to ENO1. (b) KEGG pathway analysis of differentially expressed genes related to ENO1 expression. Protein-protein interaction analysis was carried out on the genes involved in cell cycle. Note: DEGs, differentially expressed genes; KEGG, Kyoto Encyclopedia of Genes and Genomes.

The researchers obtained 1417 differentially expressed genes and 4159 genes related to ENO1 expression, overlapped all of them, and obtained 829 differentially expressed genes relevant to ENO1 expression (Figure 6(a)). These differential genes largely participated in cell division and proliferation (Supplementary Figures 9–11). Protein-protein interaction analysis was carried out on the genes involved in cell cycle (Figure 6(b)), cellular senescence, and p53 pathway, and the analysis helped researchers identify 10 core genes that were most closely associated with ENO1 protein (Supplementary Figure 12). These 10 genes all took part in the cell cycle pathway, including CHEK1, CCNB1, CHEK2, CDK1, FOXM1, E2F3, CDC25C, PTTG1, CDC45, and MCM2. According to TCGA-GTEx, these hub genes had notable correlation with the expression of ENO1 (Supplementary Figure 13).

## 4. Discussion

In our research, the upregulation of ENO1 was confirmed by in-house detections and public high-throughput data, including 1163 cases of LUSC. It was exciting to discover that a larger sample of 1860 cases indicated that the upregulated ENO1 was a risk factor in unfavorable prognosis of LUSC. ENO1 may become a new prognostic marker and therapeutic target for LUSC in the future. However, results from other six research reports varied in the expression of ENO1 in lung cancer tissues, and these studies had their limitations. For instance, Zhang et al. [12] was one of the first researchers to uncover the upregulated expression of ENO1 in 16 cases of LUSC, but the research did not cover how ENO1 mRNA was expressed in LUSC. Fu et al. [13] reported that the expression of ENO1 mRNA was elevated in 26 cases of NSCLC; however, their research failed to further explore the expression of ENO1 mRNA in LUSC tissues. Chang et al. [14] and Zhang et al. [16] unveiled that upregulated expression of ENO1 protein occurred in lung tumor tissues (80 and 72), while they offered no information regarding the expression of ENO1 protein and its mRNA in LUSC. The results of Chen et al. [15], who researched 58 cases of lung carcinoma, were consistent with the results of Chang et al. [14] and Zhang et al. [16]. Interestingly, Chang et al. [17] in 2013 reported that ENO1 protein was considerably low expressed in 46 cases of NSCLC. The study by Chang et al. [17] differed in results from other 5 studies [12–16]. It might be attributed to the differences in the race (American vs. Chinese). Besides, these studies had small sample sizes. To compensate for the insufficient sample, our study was carried out on the experimental cohorts from Guangxi, China, and revealed that the expression of ENO1 was upregulated in LUSC tissues. This finding was in accordance with the discovery by the abovementioned 5 Chinese studies [12–16].

Previously, only Zhang et al. [12] studied the protein level of ENO1 in LUSC in 2010. They reported that ENO1 was highly expressed in LUSC tissues, which was consistent with the protein level detected in our group. However, the study by Zhang et al. had a small sample size, which was acquired from only one cohort, and their research method was not diversified. Their research reflected some defects of early LUSC research, including the lack of using global resources to fully analyze the expression of ENO1 in LUSC and the ignorance of silicon methods for research into potential molecules. On the contrary, our research improved on the sample size and the research approaches. This study employed a wide range of techniques, including in-house RT-qPCR, in-house microarray, in-house immunohistochemical staining, public microarray and sequencing, and integration analysis. Also, our study involved a large sample (1163 cases of LUSC and 941 cases of non-LUSC lung controls) and verified the overexpression of ENO1 in LUSC. The combination of multiple methods and large sample data allowed the research results more valid and reliable. It was also confirmed that ENO1 might play a carcinogenic role in the tumorigenesis of LUSC.

ENO1 was abnormally highly expressed in a wide range of cancers, and its high expression was connected with the clinicopathological characteristics of patients with tumors. Studies had reported that overexpression of ENO1 was related to the clinical staging of lung cancer [16]; also, it was closely associated with the tumor size and lymph node metastasis of breast cancer [19], and it had a positive relationship with the clinical stage and lymph node metastasis of pancreatic cancer [20]. The results of this study showed that the high expression of ENO1 was linked to the T stage and clinical stage of LUSC, which were consistent with the results of other studies on tumors, suggesting that increasing the expression of ENO1 will accelerate the malignant process of LUSC.

Some scholars had different perspectives on the fact that patients with high ENO1 expression had a poor unwanted prognosis. Chinese patients with high expression of ENO1 had poor prognosis in a variety of cancers, including breast cancer [19], glioma [7], bladder cancer [9], pancreatic cancer [20], liver cancer [21], gastric cancer [33] (Chinese). However, Chang found that 46 patients with non-small cell lung cancer (NSCLC) whose expression of ENO1 was downregulated had a bad prognosis from American cohorts [17]. A number of studies varied on the prognostic capability of ENO1, which may be explained by the different races involved in the studies and the small sample sizes. In order to improve on the previous studies, we conducted an integration analysis of the prognostic statistics acquired from worldwide-accessible 17 RNA-sequencing datasets and microarrays and comprehensively evaluated the prognostic value of ENO1 in 1860 patients with LUSC. Our results indicated that patients with higher expression of ENO1 had an undesirable prognosis, suggesting that ENO1 may be applied as a new prognostic indicator for LUSC.

Overexpressed ENO1 had been observed in many tumor cells, and the upregulation of ENO1 expression might be correlated to aerobic glycolysis of cancer cells and the occurrence of malignant tumors [33]. Cancer cells largely depended on glycolysis to generate energy, and ENO1 was one of the vital enzymes that promoted cell glycolysis [5]. ENO1 played an essential role in the glycolysis process of tumor cells. Studies had found that the expression of ENO1 was related to the proliferation, invasion, and migration of tumors, including lung cancer [13, 15], gastric cancer [33], glioma [7], and pancreatic ductal glands cancer [34]. Overexpression of ENO1 could remarkably increase the proliferation, clone formation, migration, and invasion of NSCLC cells, as well as the initiation and metastasis of tumors in vivo [13]. ENO1 was highly expressed in the lung cancer cell line H1299 and stimulated the proliferation of tumor cells. In summary, the existing evidence showed that ENO1 could act as an oncogene in NSCLC and it played a part in the proliferation and metastasis of NSCLC. LUSC was a main subtype of highly aggressive non-small cell lung cancer. This study confirmed that ENO1 was highly expressed in LUSC tissues, suggesting that ENO1 may be used as a new marker for molecular targeted therapy for LUSC patients.

The interaction between ENO1 and other genes could partly reveal the underlying mechanism of ENO1 in LUSC. In this study, we evaluated the potential role of ENO1 in the initiation and development of LUSC by analyzing the functions and pathways of differential genes in LUSC and genes related to ENO1 expression. The results indicated that genes related to ENO1 expression were mainly involved in cell division and cell proliferation. Previous studies confirmed that ENO1 took part in the proliferation of tumor cells. It was speculated that ENO1 was expressed with similar gene expression patterns in different cancers, and it participated in the occurrence and development of tumors in LUSC tissues. Our pathway analysis showed that differentially expressed genes related to ENO1 expression were mainly enriched in the cell cycle and cell senescence. ENO1 could regulate NSCLC and gastric cancer by regulating the cell cycle [13, 35]. Similarly, low expression of ENO1 could regulate pancreatic ductal adenocarcinoma [34] and ovarian cancer [36] by inducing tumor cell senescence. Thus, it could be most reasonably inferred that ENO1 and the differentially expressed genes related to ENO1 expression was likely to get involved in the regulation of cell cycle and cell senescence, thereby regulating the proliferation of LUSC cells.

In order to holistically analyze the potential mechanism of ENO1 and the differential genes related to its expression in LUSC, the researchers selected 10 core genes most closely related to the role of ENO1 protein. The expression of PTTG1 in breast cancer was upregulated. Nevertheless, after silencing the PTTG1 gene, the expression of the ENO1 downstream increased, thereby regulating the proliferation and apoptosis of breast cancer cells [37]. It could be speculated that ENO1 was reversely regulated by breast cancer carcinogen PTTG1. Elemene is a new anticancer drug, which reduced the expression of CCNB1 and CDC25C by regulating the cell cycle and retarded the growth of NSCLC cells [38]. Moreover, the regulatory mechanism of elemene on the cell cycle of NSCLC relied on CHEK2 [38]. Hence, the expression of CCNB1, CDC25C, and CHEK2 played an important role in the carcinogenicity and tumor progression of NSCLC. The researchers confirmed above that ENO1 had a similar expression pattern to CCNB1, CDC25C, and CHEK2, and ENO1 might become a new targeted therapy target for LUSC. The transcription factor E2F3 bound to the QKI-6 promoter to induce the transcription of QKI-6 and then controlled the occurrence and development of bladder cancer by regulating the cell cycle [39]. There was no research on the relationship between ENO1 and QKI-6 expression, and no report on the regulatory relationship between E2F3 transcription factor and ENO1. It remained still a question whether the E2F3 transcription factor could play a role in the tumor formation and development of LUSC by binding to the ENO1 promoter, which required more sophisticated experiments for clarification. The transcription factor FOXM1 regulated the cell cycle by upregulating the expression of CENPA and CENPB and ultimately promoted the monoclonal proliferation of LUSC cells [40]. No studies have reported that FOXM1 could bind to the ENO1 promoter to stimulate the proliferation of tumor cells. After miR-195 was inhibited, it would reversely regulate the overexpression of CHEK1, thereby strengthening the resistance of NSCLC cells to microtubule-targeted drugs. Presently, no studies have reported whether ENO1 was also regulated by miR-195 and whether it functioned effectively in NSCLC targeted therapy. CDK1 was the direct target of miR-181a, which inhibited the proliferation of NSCLC cells by regulating the levels of mRNA and protein of CDK1 [41]. The regulatory relationship between miR-181a and ENO1 was not reported yet. This paper confirmed that CDK1 and ENO1 had similar and relevant expression patterns. The researchers speculated that miR-181a might regulate the expression of ENO1, thus influencing the proliferation of LUSC cells. Experiments showed that downregulation of CDC45 expression would block the cell cycle and thereby suppressed the proliferation of NSCLC cells [42]. Nonetheless, no studies have focused on the specific molecular mechanism of CDC45 in NSCLC, which had a similar expression pattern to ENO1. The high expression of UBA2 would simultaneously promote the expression of MCM2 and speed up the proliferation of NSCLC cells by regulating the cell cycle [43]. However, there have been no studies regarding the specific molecular mechanism of UBA2 in NSCLC and whether the expression of ENO1 was regulated by UBA2.

In summary, the expression profiles of ENO1 and its core genes bore similarities. Therefore, the researchers could infer the potential mechanism of ENO1 in LUSC by appraising the known mechanism of the hub genes associated with LUSC.

This study applied evidence-based concepts to probe into the prognostic value, expression significance, and potential mechanism of ENO1 in LUSC, but there were still limitations. The heterogeneity existed in the ENO1 expression datasets. Single-cell RNA-seq of LUSC tissues has not been performed to confirm the clinical significance of ENO1. Also, cell function experiments were not performed to verify the molecular mechanism of ENO1 in LUSC. In the future, we will design precise and rigorous experiments to verify the conclusions of this study.

## 5. Conclusion

This study used a variety of detection approaches and large sample sizes to confirm the abnormal upregulation of ENO1 mRNA and protein in LUSC tissues. Patients with high levels of ENO1 expression had a poorer prognosis. The increased expression of ENO1 might be affected by some of its coexpressed genes, and thus the mechanism to regulate the proliferation and invasion of LUSC cells started to exist. ENO1 has the potential to become a new prognostic marker and a new therapeutic target for LUSC. We would conduct a series of in vitro and in vivo experiments to confirm this hypothesis.

## Figures and Tables

**Table 1:** The primer sequences of ENO1 and ACTB.

	Primer	Sequence
Internal reference ACTB	Forward primer	TCTTCGCCTTAATACTTGT
	Reverse primer	AAGCCTTCATACATCTCAA

ENO1	Forward primer	GTACCGCCACATCGCTGACTTG
	Reverse primer	AGCATGAGAACCGCCATTGATGAC

Note: ENO1, alpha-enolase. ACTB, actin beta.

## Data Availability

The data set of the public database can be found from the following websites: Genotype-Tissue Expression: http://genome.ucsc.edu/; The Cancer Genome Atlas database: http://genome.ucsc.edu/; Gene Expression Omnibus: https://www.ncbi.nlm.nih.gov/geo/query/acc.cgi; ArrayExpress: https://www.ebi.ac.uk/arrayexpress/.

## Supplementary Materials

Supplementary Materials Supplementary Figure 1: the flow chart of this research. Note: RT-qPCR, real-time quantitative polymerase chain reaction; GO, Gene Ontology; KEGG, Kyoto Encyclopedia of Genes and Genomes; PPI, protein-protein interaction network. Supplementary Figure 2: process of including public database datasets. Note: GTEx, Genotype-Tissue Expression; TCGA, The Cancer Genome Atlas database; GEO, Gene Expression Omnibus; GO, Gene Ontology; KEGG, Kyoto Encyclopedia of Genes and Genomes; PPI, protein-protein interaction network. Supplementary Figure 3: the scatter plots and ROC curves of the publicly released datasets. Note: ROC, receiver operating characteristic curve. Supplementary Figure 4: the scatter plots and ROC curves of the publicly released datasets. Note: ROC, receiver operating characteristic curve. Supplementary Figure 5: SROC diagram of ENO1 expression in LUSC. ENO1 can distinguish LUSC from normal lung tissue (AUC = 0.8705). Note: SROC, summary receiver operating characteristic curve; AUC, area under the curve; LUSC, squamous cell carcinoma of lung. Supplementary Figure 6: the integration analysis of ENO1 expression in LUSC (summary sensitivity = 0.88 [0.83, 0.92]). Supplementary Figure 7: integration analysis of ENO1 expression in LUSC (summary specificity = 0.89 [0.84, 0.94]). Supplementary Figure 8: the funnel plots of integration analysis. Supplementary Figure 9: GO function analysis of differentially expressed genes related to ENO1 expression from the perspective of CC function. Note: GO, Gene Ontology; CC, cellular component. Supplementary Figure 10: GO function analysis of differentially expressed genes related to ENO1 expression from the perspective of MF. Note: GO, Gene Ontology; MF, molecular function. Supplementary Figure 11: GO function analysis of differentially expressed genes related to ENO1 expression from the perspective of BP. Note: GO, Gene Ontology; BP, biological process. Supplementary Figure 12: core genes related to ENO1 expression. Supplementary Figure 13: correlation analysis between ENO1 and core genes. Supplementary Table 1: basic information of LUSC mRNA datasets. Supplementary Table 2: the relationship between ENO1 expression level and clinicopathological parameters in 12 patients with LUSC in our hospital. Note: M, mean; SD, standard deviation; *P*, *P* value.
