# Supplementary material for: The Indication of Poor Prognosis by High Expression of ENO1 in Squamous Cell Carcinoma of the Lung
Source: J Oncol. 2021 Aug 30;2021:9910962. doi: 10.1155/2021/9910962 (PMC8423576; doi:10.1155/2021/9910962)
Supplement: Supplementary Materials — Supplementary Figure 1: the flow chart of this research. Note: RT-qPCR, real-time quantitative polymerase chain reaction; GO, Gene Ontology; KEGG, Kyoto Encyclopedia of Genes and Genomes; PPI, protein-protein interaction network. Supplementary Figure 2: process of including public database datasets. Note: GTEx, Genotype-Tissue Expression; TCGA, The Cancer Genome Atlas database; GEO, Gene Expression Omnibus; GO, Gene Ontology; KEGG, Kyoto Encyclopedia of Genes and Genomes; PPI, protein-protein interaction network. Supplementary Figure 3: the scatter plots and ROC curves of the publicly released datasets. Note: ROC, receiver operating characteristic curve. Supplementary Figure 4: the scatter plots and ROC curves of the publicly released datasets. Note: ROC, receiver operating characteristic curve. Supplementary Figure 5: SROC diagram of ENO1 expression in LUSC. ENO1 can distinguish LUSC from normal lung tissue (AUC = 0.8705). Note: SROC, summary receiver operating characteristic curve; AUC, area under the curve; LUSC, squamous cell carcinoma of lung. Supplementary Figure 6: the integration analysis of ENO1 expression in LUSC (summary sensitivity = 0.88 [0.83, 0.92]). Supplementary Figure 7: integration analysis of ENO1 expression in LUSC (summary specificity = 0.89 [0.84, 0.94]). Supplementary Figure 8: the funnel plots of integration analysis. Supplementary Figure 9: GO function analysis of differentially expressed genes related to ENO1 expression from the perspective of CC function. Note: GO, Gene Ontology; CC, cellular component. Supplementary Figure 10: GO function analysis of differentially expressed genes related to ENO1 expression from the perspective of MF. Note: GO, Gene Ontology; MF, molecular function. Supplementary Figure 11: GO function analysis of differentially expressed genes related to ENO1 expression from the perspective of BP. Note: GO, Gene Ontology; BP, biological process. Supplementary Figure 12: core genes related to ENO1 expression. Supplementa [file 9910962.f1.docx]

**Supplementary Figures and Tables**


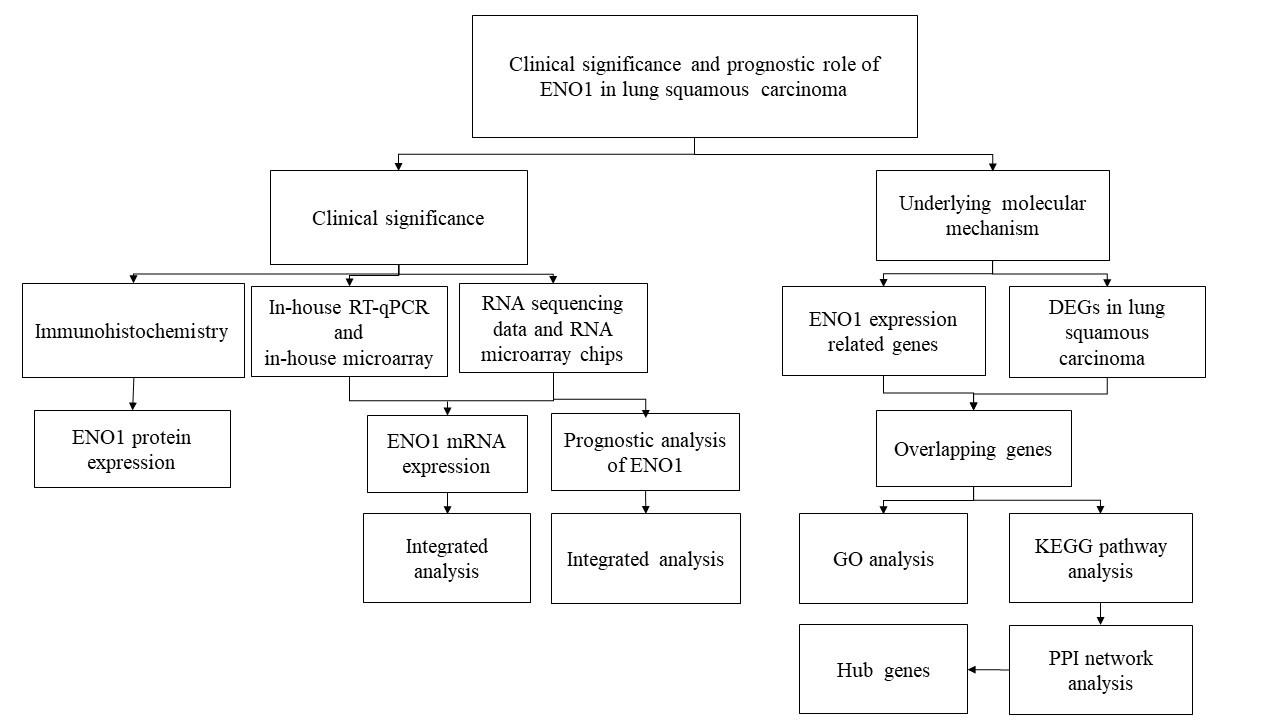


Supplementary Figure 1 The flow chart of this research.

Note: RT-qPCR, real-time quantitative polymerasechain reaction; Go, Gene Ontology; KEGG, Kyoto Encyclopedia of Genes and Genomes; PPI, protein-protein interaction network.


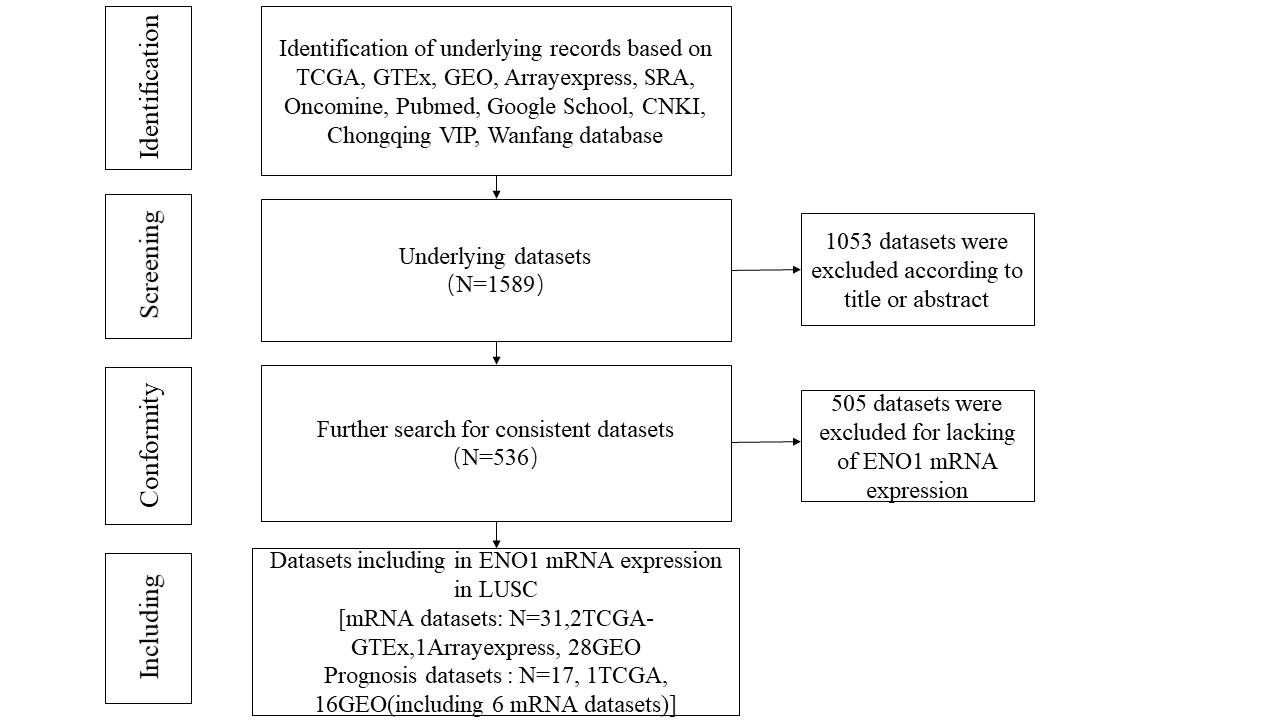


Supplementary Figure 2 Process of including public database datasets.

Note: GTEx,Genotype-Tissue Expression; TCGA, The Cancer Genome Atlas database; GEO, Gene Expression Omnibus; GO, Gene Ontology; KEGG, Kyoto Encyclopedia of Genes and Genomes; PPI, protein-protein interaction network.


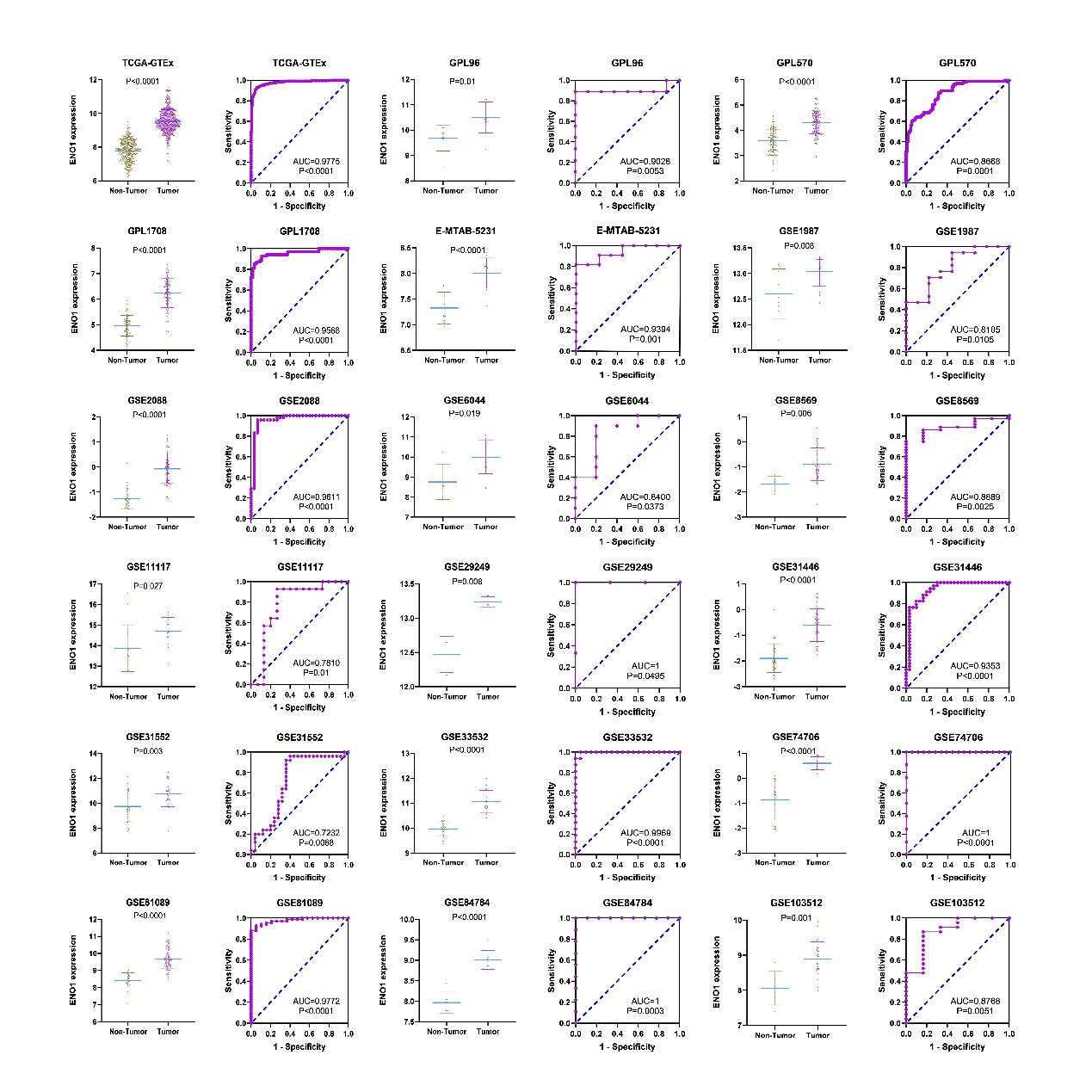


Supplementary Figure 3 The scatter plots and ROC curves of the publicly released datasets.

Note: ROC, receiver operating characteristic curve.


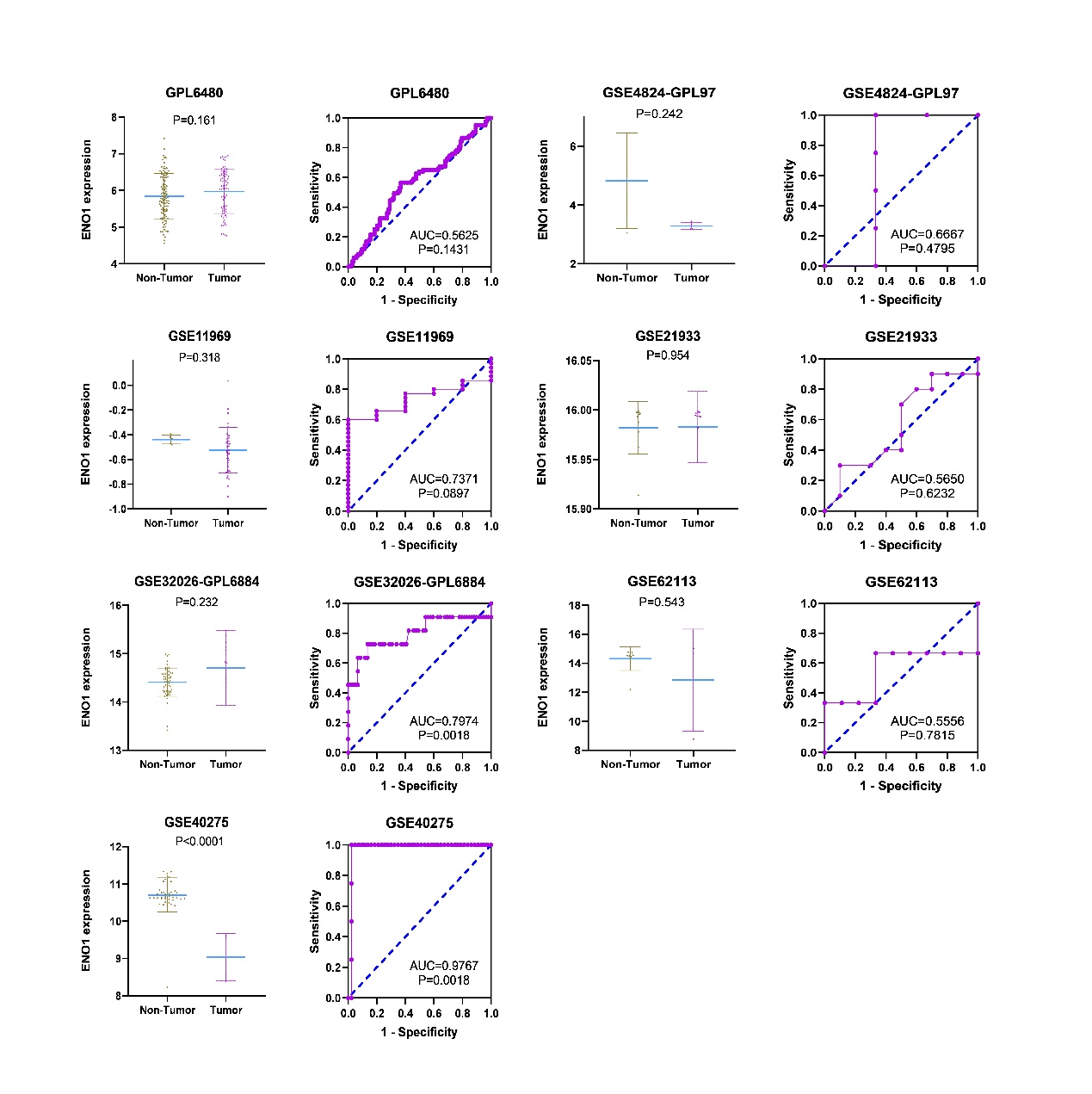


Supplementary Figure 4 The scatter plots and ROC curves of the publicly released datasets.

Note: ROC, receiver operating characteristic curve.


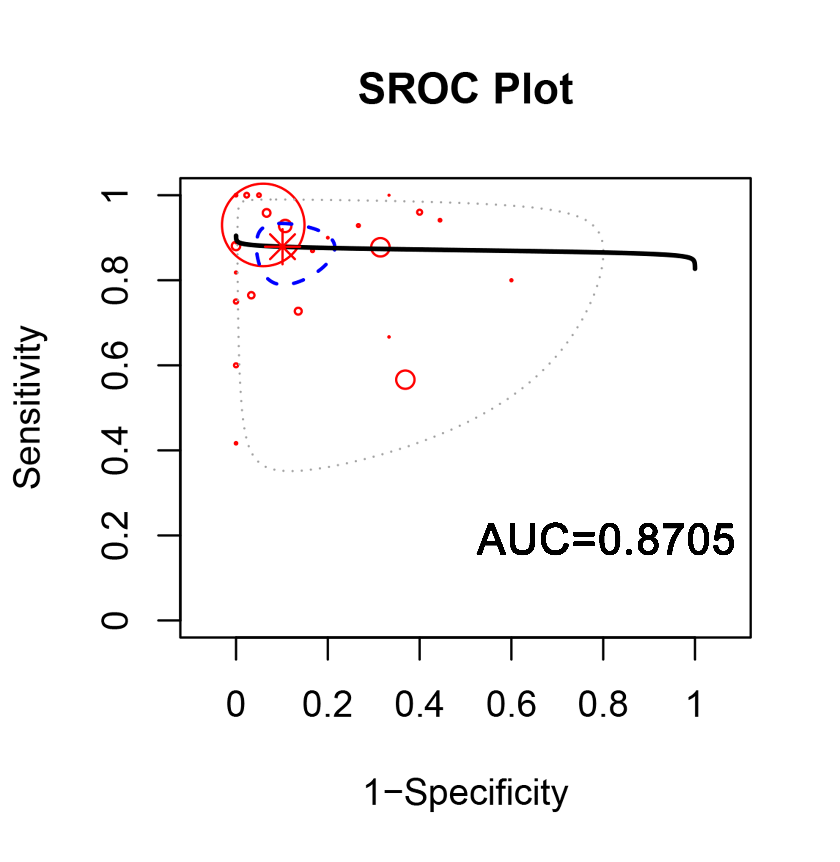


Supplementary Figure 5 SROC diagram of ENO1 expression in LUSC. ENO1 can distinguish LUSC from normal lung tissue (AUC=0.8705).

Note: SROC, summary receiver operating characteristic curve; AUC, area under the curve; LUSC, squamous cell carcinoma of lung.


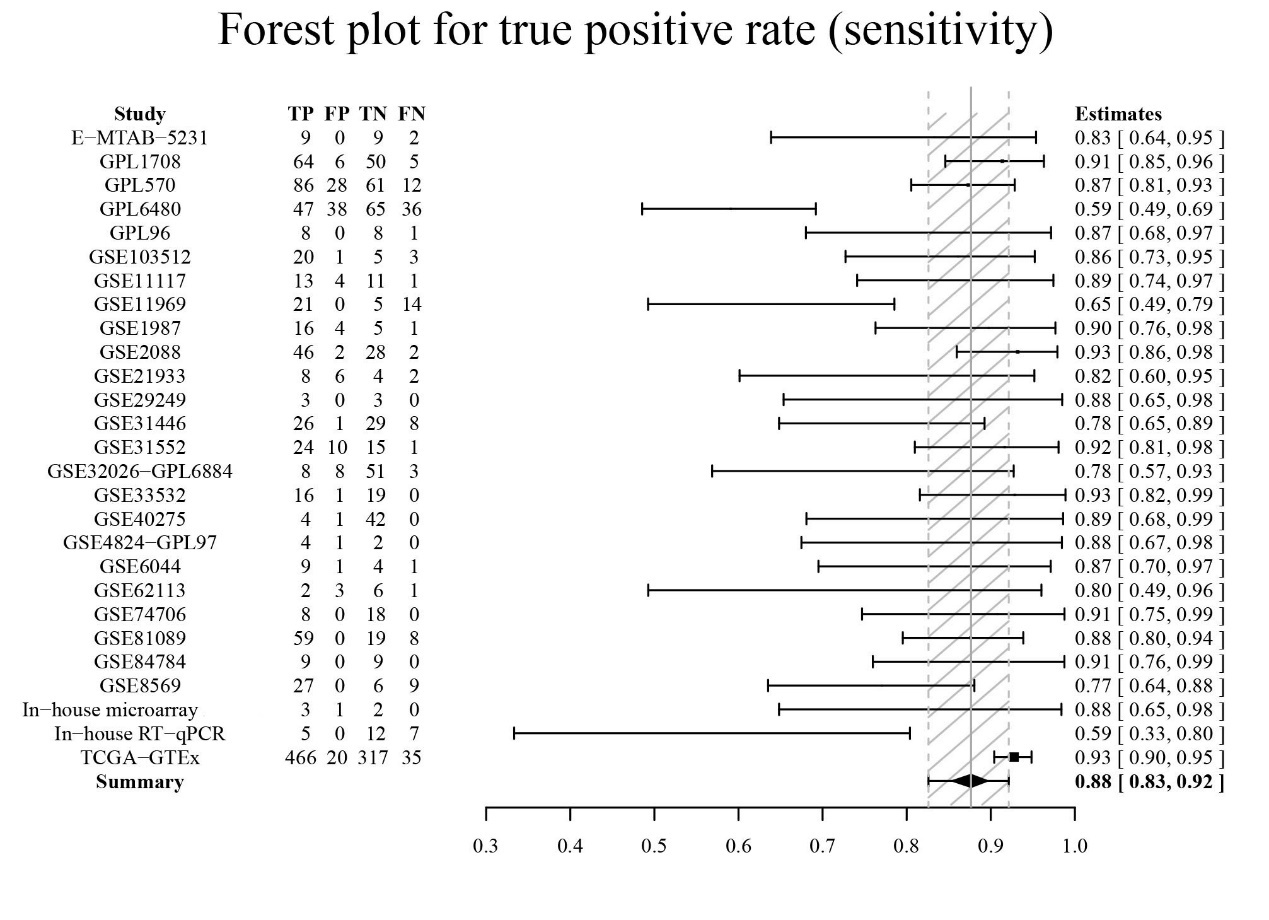


Supplementary Figure 6 The Integration-analysis of ENO1 expression in LUSC (summary sensitivity=0.88[0.83, 0.92]).


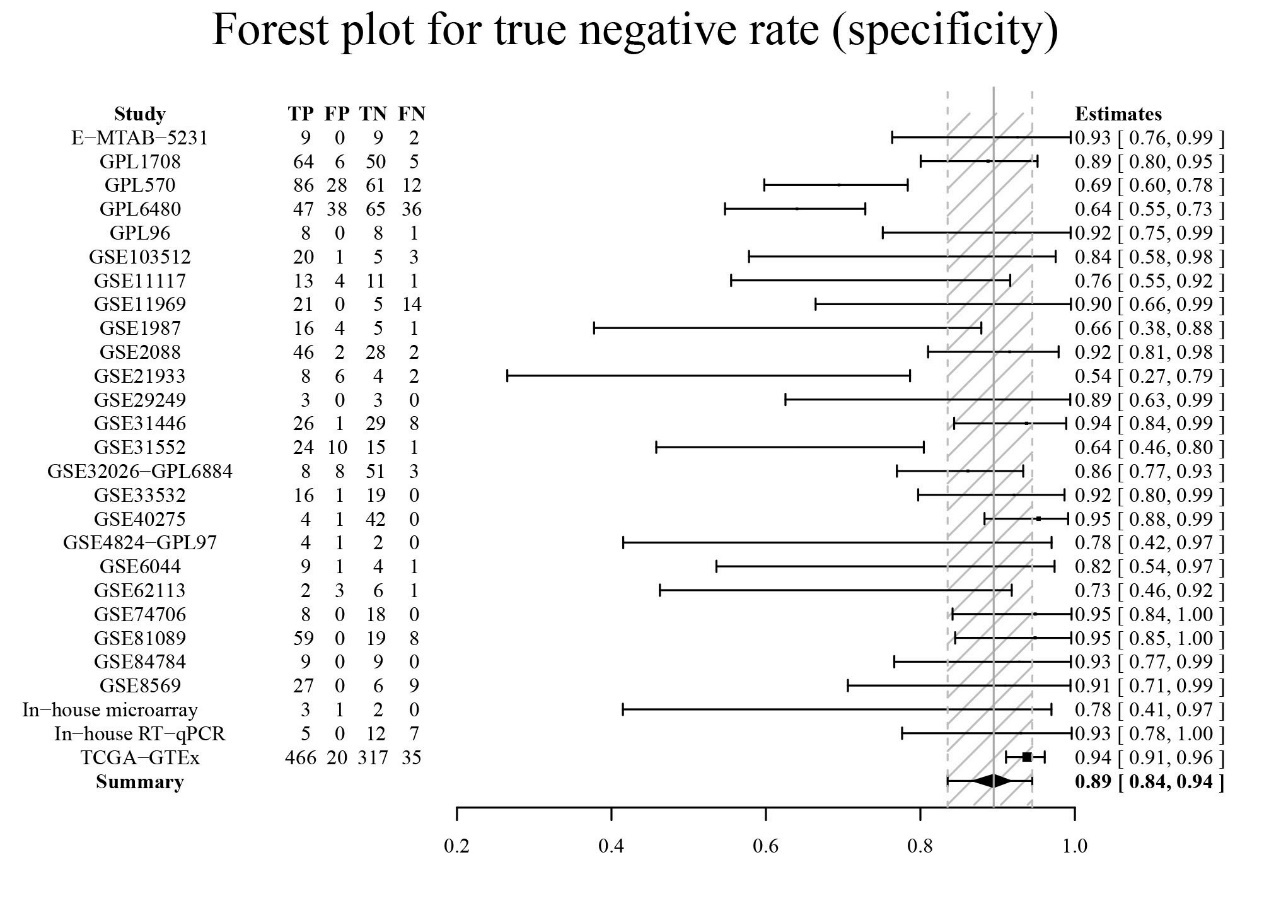


Supplementary Figure 7 Integration-analysis of ENO1 expression in LUSC (summary specificity=0.89[0.84, 0.94])


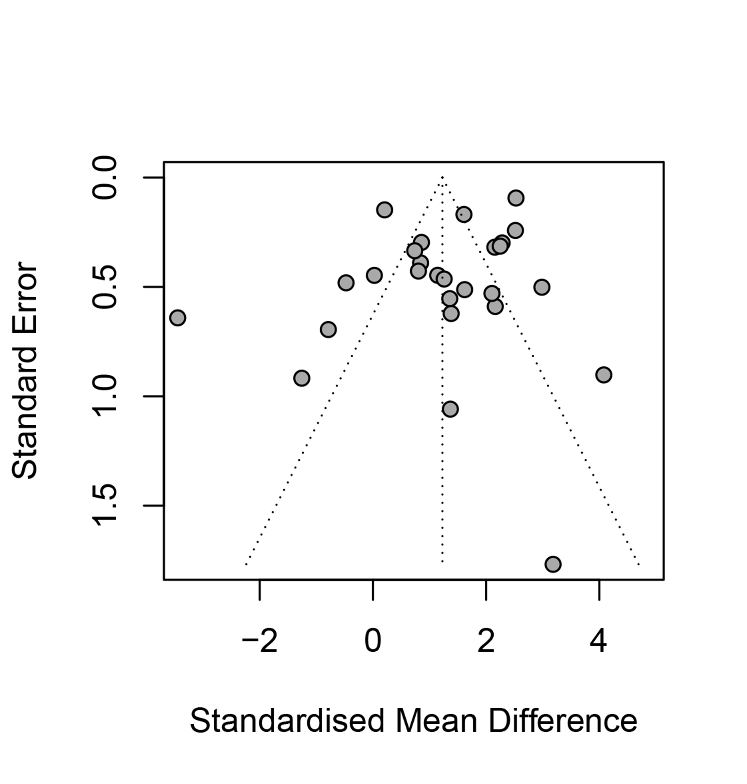


Supplementary Figure 8 The funnel plots of Integration-analysis.


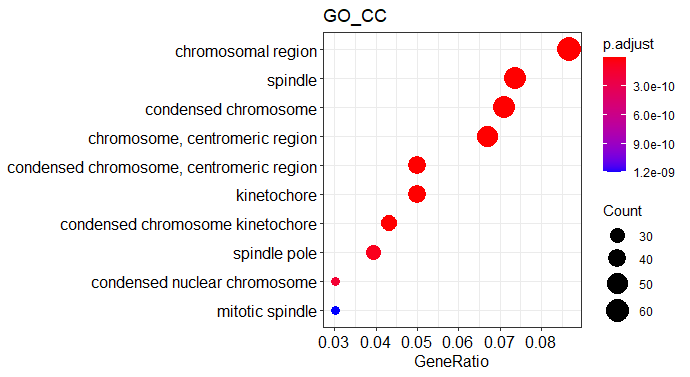


Supplementary Figure 9 GO function analysis of differentially expressed genes related to ENO1 expression from the perspective of CC function.

Note: GO, Gene Ontology; CC, cellular component.


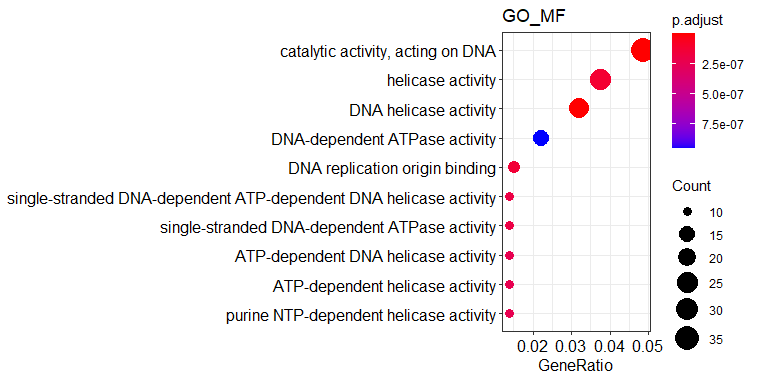


Supplementary Figure 10 GO function analysis of differentially expressed genes related to ENO1 expression from the perspective of MF.

Note: GO, Gene Ontology; MF, molecular function.


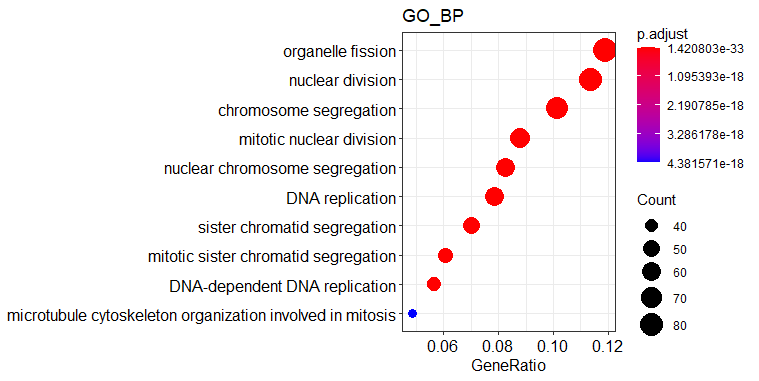


Supplementary Figure 11 GO function analysis of differentially expressed genes related to ENO1 expression from the perspective of BP.

Note: GO, Gene Ontology; BP, biological process.


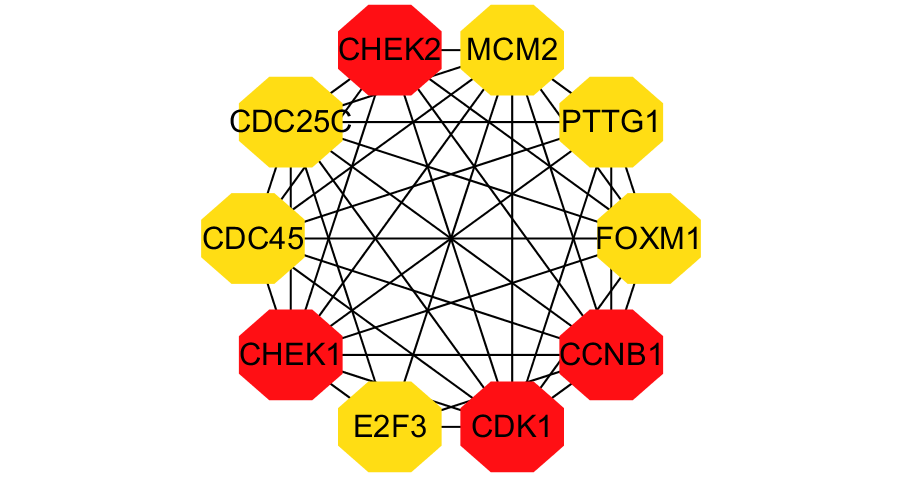


Supplementary Figure 12 Core genes related to ENO1 expression.


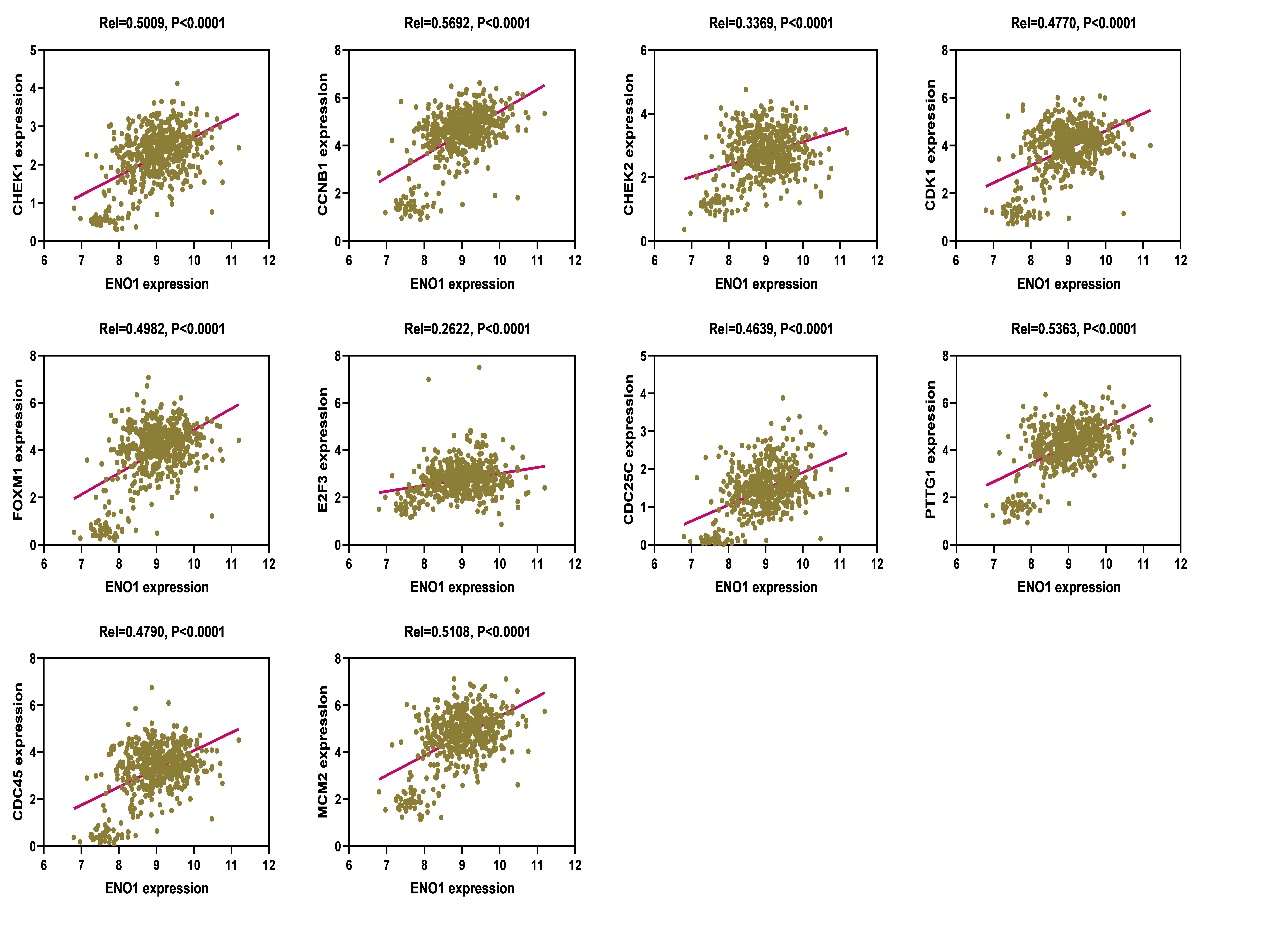


Supplementary Figure 13 Correlation analysis between ENO1 and core genes

Supplementary Table 1 Basic information of LUSC mRNA datasets.

| Study | Transcription profiling | Year | Platform | Tumor | Control |
| --- | --- | --- | --- | --- | --- |
| E-MTAB-5231 | Microarray | 2017 | / | 11 | 9 |
| GSE103512 | Microarray | 2017 | GPL13158 | 23 | 6 |
| GSE11117 | Microarray | 2008 | GPL6650 | 14 | 15 |
| GSE11969 | Microarray | 2008 | GPL7015 | 35 | 5 |
| GSE12428 | Microarray | 2008 | GPL1708 | 34 | 28 |
| GSE12472 | Microarray | 2008 | GPL1708 | 35 | 28 |
| GSE19188 | Microarray | 2010 | GPL570 | 27 | 65 |
| GSE1987 | Microarray | 2004 | GPL91 | 17 | 9 |
| GSE2088 | Microarray | 2004 | GPL962 | 48 | 30 |
| GSE21933 | Microarray | 2010 | GPL6254 | 10 | 10 |
| GSE27489 | Microarray | 2011 | GPL570 | 10 | 10 |
| GSE29249 | Microarray | 2011 | GPL10558 | 3 | 3 |
| GSE30219 | Microarray | 2011 | GPL570 | 61 | 14 |
| GSE31446 | Microarray | 2011 | GPL9244 | 34 | 30 |
| GSE31552 | Microarray | 2011 | GPL6244 | 25 | 25 |
| GSE32026 | Microarray | 2011 | GPL4133 | 11 | 59 |
| GSE3268 | Microarray | 2005 | GPL96 | 5 | 5 |
| GSE33479 | Microarray | 2014 | GPL6480 | 14 | 95 |
| GSE33532 | Microarray | 2014 | GPL572 | 16 | 20 |
| GSE40275 | Microarray | 2012 | GPL15974 | 4 | 43 |
| GSE4824 | Microarray | 2006 | GPL96 | 4 | 3 |
| GSE4824 | Microarray | 2006 | GPL97 | 4 | 3 |
| GSE6044 | Microarray | 2006 | GPL201 | 10 | 5 |
| GSE62113 | Microarray | 2014 | GPL14951 | 3 | 9 |
| GSE67061 | Microarray | 2016 | GPL6480 | 69 | 8 |
| GSE74706 | Microarray | 2016 | GPL13497 | 8 | 18 |
| GSE81089 | High throughput sequencing | 2016 | GPL16791 | 67 | 19 |
| GSE84784 | Microarray | 2019 | GPL17585 | 9 | 9 |
| GSE8569 | Microarray | 2007 | GPL5645 | 36 | 6 |
| In-house microarray matrix | Microarray | 2020 | / | 3 | 3 |
| In-house RT-qPCR | PCR | 2019 | / | 12 | 12 |
| TCGA-GTEx | High throughput sequencing | 2020 | / | 501 | 337 |

Supplementary Table 2 The relationship between ENO1 expression level and clinicopathological parameters in 12 patients with LUSC in our hospital.

| Clinical Parameters | |  | N |  | M±SD | P |
| --- | --- | --- | --- | --- | --- | --- |
| Age |  | |  |  |  |  |
|  | <60years | | 5 |  | 1.2484±1.5472 | 0.504 |
|  | ≥60years | | 7 |  | 1.7729±1.0895 |  |
| Gender |  | |  |  |  |  |
|  | Male | | 9 |  | 1.7767±1.3844 | 0.122 |
|  | Female | | 3 |  | 0.8873±0.44 |  |
| Stage |  | |  |  |  |  |
|  | Stage1-Stage2 | | 9 |  | 1.8727±1.3109 | 0.021 |
|  | Stage3-Stage4 | | 3 |  | 0.5993±0.2255 |  |
| T |  | |  |  |  |  |
|  | T1-T2 | | 9 |  | 1.8681±1.3149 | 0.023 |
|  | T3-T4 | | 3 |  | 0.6132±0.2495 |  |
| N |  | |  |  |  |  |
|  | N0 | | 8 |  | 1.7283±1.3227 | 0.525 |
|  | N1 | | 4 |  | 1.2065±1.2284 |  |
| M |  | |  |  |  |  |
|  | M0 | | 11 |  | 1.6555±1.2707 | / |
|  | M1 | | 1 |  | 0.4414/ |  |

Note: M, mean; SD, standard deviation; P, p value.
